# Epigenetic regulation of ecotype-specific expression of the heat-activated transposon *ONSEN*

**DOI:** 10.3389/fpls.2022.899105

**Published:** 2022-07-18

**Authors:** Kosuke Nozawa, Seiji Masuda, Hidetoshi Saze, Yoko Ikeda, Takamasa Suzuki, Hiroki Takagi, Keisuke Tanaka, Naohiko Ohama, Xiaoying Niu, Atsushi Kato, Hidetaka Ito

**Affiliations:** ^1^Graduate School of Life Sciences, Hokkaido University, Sapporo, Japan; ^2^Plant Epigenetics Unit, Okinawa Institute of Science and Technology, Onna-son, Japan; ^3^Institute of Plant Science and Resources, Okayama University, Kurashiki, Japan; ^4^College of Bioscience and Biotechnology, Chubu University, Kasugai, Japan; ^5^Faculty of Bioresources and Environmental Sciences, Ishikawa Prefectural University, Nonoichi, Japan; ^6^NODAI Genome Research Center, Tokyo University of Agriculture, Setagaya-ku, Japan; ^7^Graduate School of Agricultural and Life Sciences, The University of Tokyo, Bunkyo-ku, Japan; ^8^Faculty of Science, Hokkaido University, Sapporo, Japan

**Keywords:** transposons, ecotype, environmental stress, *Arabidopsis thaliana*, *ONSEN*

## Abstract

Transposable elements are present in a wide variety of organisms; however, our understanding of the diversity of mechanisms involved in their activation is incomplete. In this study, we analyzed the transcriptional activation of the *ONSEN* retrotransposon, which is activated by high-temperature stress in *Arabidopsis thaliana*. We found that its transcription is significantly higher in the Japanese ecotype Kyoto. Considering that transposons are epigenetically regulated, DNA methylation levels were analyzed, revealing that CHH methylation was reduced in Kyoto compared to the standard ecotype, Col-0. A mutation was also detected in the Kyoto *CMT2* gene, encoding a CHH methyltransferase, suggesting that it may be responsible for increased expression of *ONSEN*. CHH methylation is controlled by histone modifications through a self-reinforcing loop between DNA methyltransferase and histone methyltransferase. Analysis of these modifications revealed that the level of H3K9me2, a repressive histone marker for gene expression, was lower in Kyoto than in Col-0. The level of another repressive histone marker, H3K27me1, was decreased in Kyoto; however, it was not impacted in a Col-0 *cmt2* mutant. Therefore, in addition to the *CMT2* mutation, other factors may reduce repressive histone modifications in Kyoto.

## Introduction

Mobile elements or transposable elements (TEs) are abundant in the genomes of many organisms (Bucher et al., 2012; Fultz et al., 2015). Their high copy numbers suggest that they are actively transposed in the genome; however, at higher rates of transposition, they can disrupt key genes and thus decrease survival. As such, hosts control transposon activation *via* epigenetic silencing mechanisms, including DNA methylation and histone modification. Several important factors have been identified for DNA methylation in *Arabidopsis thaliana*. For example, the DNA methyltransferase MET1 methylates cytosines in CG contexts, whereas CMT2 is responsible for CHH methylation, and CMT3 is responsible for CHG methylation (H represents A, T, or C bases) (Du et al., 2012; Stroud et al., 2014; Zhang et al., 2018). Non-CG methylation is maintained through a feedback loop between DNA methylation and histone modification.

In plants, RNA-directed DNA methylation (RdDM) is an important *de novo* methylation pathway (Wierzbicki et al., 2008; Gao et al., 2010). The plant-specific RNA polymerase IV transcribes TEs. Single-stranded RNA is converted into double-stranded RNA by RNA-dependent RNA polymerase II. Dicer cleaves double-stranded RNA into short dsRNA fragments called small RNAs, which are incorporated into the Argonaute protein (AGO) complex and transported to the target sequence. The AGO complex binds to the target RNA transcribed by RNA polymerase V, another plant-specific RNA polymerase, based on the small RNA sequence in the AGO complex, which is then accessed by the methyltransferase DRM2. The target sequence is subsequently subjected to *de novo* methylation *via* DRM2 (Cao and Jacobsen, 2002; Matzke and Birchler, 2005). By this mechanism, ectopically activation of TE expression results in DNA methylation of complementary sequences and transcriptional repression.

Most TEs are inactivated by epigenetic silencing mechanisms; however, some are activated by environmental stress (Lisch, 2009; Naito et al., 2009). These stress-activated transposons in plants contain stress-responsive *cis*-sequences in their promoter regions and exhibit transcriptional activity similar to that of stress-responsive genes. One such example is the Ty1/*copia*-like retrotransposon *ATCOPIA78*/*ONSEN* of *A. thaliana*: it is activated by high-temperature stress (Ito et al., 2011). *ONSEN* has a heat response element sequence in its long terminal repeat that binds to heat shock factor A2 (HsfA2), which activates transcription at high temperatures (Cavrak et al., 2014). *ONSEN* is more strongly upregulated in mutants deficient in the RdDM pathway than in wild-type (WT) plants (Ito et al., 2011). In addition, a high frequency of new *ONSEN* insertions has been observed in the progeny of stressed plants deficient in siRNA biogenesis (Ito et al., 2016). *ONSEN* expression has also been observed in heat-stressed WT and other epigenetic mutants; however, transgenerational transposition was absent in their progeny (Ito et al., 2011). These results suggest that small RNA-mediated regulation participates in *ONSEN* transcriptional and transpositional control.

There are eight copies of *ONSEN* in the genome of the *A. thaliana* Columbia (Col-0) ecotype. *ONSEN* preferentially targets the euchromatic regions and affects gene expression in Col-0 (Ito et al., 2016). For example, *ONSEN* insertion into the abscisic acid (ABA)-responsive gene *ABI4* results in an ABA-insensitive phenotype (Ito et al., 2016). Although *ONSEN* copy numbers vary within the same species (Ito et al., 2013), the genomic locations and copy numbers vary among *Arabidopsis* ecotypes (Masuda et al., 2017). Therefore, the purpose of the current study is to compare the transcription levels of *ONSEN* under heat stress in *Arabidopsis* ecotypes—including Col-0 and the Japanese ecotype, Kyoto—to better understand *ONSEN* transcriptional regulation. In particular, regulatory mechanisms were analyzed from an epigenetic perspective. Finally, genome-wide gene expression, DNA methylation, and histone modification analyses were conducted to understand the epigenetic traits of *Arabidopsis* ecotype Kyoto.

## Materials And Methods

### Plants and growing conditions

Seeds of *Arabidopsis* ecotype Kyoto (CS76535) and *cmt2* (CS849188) of the Col-0 background, were ordered from the ABRC Stock Center (The Ohio State University, Columbus, OH, United States). Seeds of Japanese ecotypes FK (SJW11300), TY (SJW11800), IK (SJW12100), AB (SJW12200), NG (SJW12500), YGA (SJW11900), Kyoto-1 (SJW13700), Kyoto-2 (SJW13800), Kyoto-3 (SJW13900), Hir-1 (SJW10200), Shoke2i (SJW10100), Yam-1 (SJW10401), Yam-2 (SJW10402), Izumo (SJW11100), TO (SJW11200), SK (SJW11400), YGU (SJW11500), Fuk (SJW11600), ES (SJW11700), TKS (SJW12000), OY (SJW12300), Enoshima (SJW12600), Sap-0 (SJW12800), Sap-1 (SJW12900), Sap-2 (SJW13000), Eniwa (SJW13300), Sendai-1 (SJW13100), Sendai-2 (SJW13200), Sendai-3 (SJW13500), Sendai-4 (SJW14400), and RIBI (SJW14300) were purchased from The SENDAI *Arabidopsis* Seed Stock Center (Miyagi University of Education, Sendai, Japan). Seeds of Ws-2, Ler, and Ts were obtained from the laboratory of Kazuko Shinozaki (University of Tokyo). Plants were grown on Murashige and Skoog (MS) plates containing 0.1% sucrose and 0.8% agar, or in culture soil (vermiculite:soil = 6:1) under continuous light at 21°C.

### Stress treatments

Seven-day-old germinated *Arabidopsis* plants sown on MS plates at 21°C were transferred to a 37°C incubator. For expression analysis after heat treatment, plants exposed to 37°C for 24h were sampled immediately following heat treatment and RNA was extracted. For thermotolerance testing, plants were grown on agar plates containing half-strength MS medium for 6 days in a growth chamber at 22°C. After sealing with Parafilm M, plates were submerged in a water bath at 43°C for 35–55 min, returned to the growth chamber, and incubated for 8 days to allow recovery. Thermotolerance was evaluated by bleaching and generation of new leaves after recovery.

### Southern blotting analysis

Genomic DNA of was isolated using a Nucleon PhytoPure DNA extraction kit (Cytiva RPN8511). Southern blots were performed as previously described (Miura et al., 2004). Hybridization was detected using the Megaprime DNA Labeling System (Cytiva GERPN1607) and high SDS hybridization buffer with radioactive *ONSEN*-specific probes.

### Real-time polymerase chain reaction

Total RNA was extracted from seedlings using TRI reagent (Sigma-Aldrich 93289), according to the supplier’s recommendations. Five plants were pooled before RNA extraction. Approximately 3–5 μg of total RNA was treated with RQ1 RNase-free DNase (Promega M6101) and reverse transcribed using the ReverTraAce qPCR RT Kit (TOYOBO FSQ-101). To quantify *ONSEN* DNA, genomic DNA was extracted from seedlings using the Nucleon PhytoPure DNA extraction kit (Cytiva RPN8511). An Applied Biosystems 7300 Real-Time PCR System was used to quantify *ONSEN* DNA using the THUNDERBIRD SYBR qPCR Mix (TOYOBO QPS-201). Each experiment (with three biological and technical replicates) was performed in triplicate.

### Mapping

Kyoto was first crossed with Col-0 and then self-pollinated to produce a segregating F2 population of the Kyoto line. After heat stressing seedlings at 37°C for 24 h from the F3 population that self-pollinated the F2 population, *ONSEN* was quantified by qRT-PCR to prepare approximately 40 lines each with high and low *ONSEN* transcript levels. Their parental DNA was prepared and subjected to next-generation sequencing. Genomic DNA was extracted using the Nucleon PhytoPure DNA Extraction Kit (GE Healthcare), fragmented by sonication (S220, Covaris), and used to construct a library using TruSeq DNA Library Prep Kits (Illumina) according to the manufacturer’s protocol. The library was sequenced by NextSeq500 (Illumina). The output bcl files were converted to fastq files by bcl2fastq (Illumina). Reads were analyzed by Mitsucal (Suzuki et al., 2018).

### Cytology

Young leaves were fixed with ethanol:acetic acid (3:1) and stored at 4°C overnight. Leaves were washed twice with distilled water for 5 min each, followed by a 5-min wash with 10 mM sodium citrate, pH 4.8. Cellulase (Yakult Cellulase Onozuka R-10), pectolyase (Kyowa Chemical Products YAKPECT23-5), and cytohelicase (Sigma-Aldrich C8274) were then added at 1% (w/v) each to citrate buffer and incubated at 37°C for 1 h. After digestion, individual leaves were placed on a slide, dissected, and disrupted using a needle in 5 μL 45% acetic acid. The slide was placed on a heat plate at 45°C for 30 s and subsequently put a drop of fixing solution, dried at 21°C. The composition of the probe mixture per slide is as follows: 2 μL DIG-labeled centromere DNA (PCR product), 2 μL 50% dextran sulfate, 2 μL 20XSSC, 10 μL formamide, and 4 μL ultrapure water. The probe mixture was denatured at 98°C for 10 min, put on a slide, denatured with the slide at 80°C for 2 min, and incubated overnight at 37°C in a moist chamber with a cover glass. The slide was washed twice with 2xSSC for 5 min. 20 μL of antibody (VEC DI-7594 Anti-DIG) was added, covered with Parafilm, and incubated in a moist chamber at 37°C for 2 h. The slide was washed twice with 2xSSC for 5 min, allowed to dry at 21°C, and counterstained with 5 μL DAPI covering with a cover glass.

### Methylation inhibition

The reagent 5-aza-2’-deoxycytidine (5AzaC; Tokyo Chemical Industry A2033) was dissolved in water and added to the MS plate at a final concentration of 0.01 mM.

### Chromatin immunoprecipitation

Chromatin immunoprecipitation (ChIP) was performed using 1–2 g of 10-day-old seedlings crosslinked with 1% formaldehyde for 20 min at 21°C. Crosslinking was quenched by the addition of glycine to a final concentration of 125 mM. Crosslinked tissues were ground in liquid nitrogen, resuspended in 20 mL of extraction buffer 1 (0.4 M sucrose, 10 mM Tris-HCl pH 8.0, 5 mM β-mercaptoethanol, and protease inhibitor cocktail tablet), and filtered through Miracloth (Millipore 475855). Nuclei were pelleted by centrifuging at 13,000 × *g* for 20 min at 4°C. Nuclei were washed with extraction buffer 2 (0.25 M sucrose, 10 mM Tris-HCl pH 8.0, 10 mM MgCl_2_, 1% Triton X-100, 5 mM 2-mercaptoethanol, and protease inhibitor cocktail tablet), resuspended in 0.6 mL of extraction buffer 2, layered onto 600 μL of extraction buffer 3 (1.7 M sucrose, 10 mM Tris-HCl pH 8.0, 0.15% Triton X-100, 2 mM MgCl_2_, 5 mM 2-mercaptoethanol, and protease inhibitor cocktail tablet) and centrifuged at 13,000 × *g* for 1 h at 4°C. The chromatin precipitate was resuspended in 300 μL SDS lysis buffer (50 mM Tris-HCl pH 8.0, 10 mM EDTA, 1% SDS, and protease inhibitor cocktail tablet).

Chromatin shearing was performed using a Bioruptor (Diagenode UCD-300) for eight cycles of 30 s “ON” and 30 s “OFF.” After centrifugation at 13,000 × *g* at 4°C for 10 min, 50 μL of the supernatant was diluted with ChIP dilution buffer (1.1% Triton X-100, 1.2 mM EDTA, 16.7 mM Tris-HCl pH 8.0, 167 mM NaCl, and protease inhibitor cocktail tablet) to a volume of 500 μL, and incubated with 3 μg of H3K9me2 antibody (Abcam, Cat#ab1220) or H3K27me1 antibody (Millipore Cat#07-448) overnight. Immune complexes were captured by incubation with 25 μL of Dynabeads Protein G (Invitrogen) for 2 h. After sequential washes with low-salt buffer (150 mM NaCl, 0.1% SDS, 1% Triton X-100, 2 mM EDTA, and 20 mM Tris-HCl pH 8), high-salt buffer (500 mM NaCl, 0.1% SDS, 1% Triton X-100, 2 mM EDTA, and 20 mM Tris-HCl pH 8), LiCl buffer (0.25 M LiCl, 1% NP-40, 1% sodium deoxycholate, 1 mM EDTA, and 10 mM Tris-HCl pH 8), and TE buffer (10 mM Tris-HCl pH 8, 1 mM EDTA), immune complexes were eluted with 10 mM Tris-HCl pH 8.0, 0.2 M NaCl, 5 mM EDTA, and 0.5% SDS, and crosslinking was reversed by incubating at 65°C for 6 h. After proteinase K and RNase treatment, DNA was purified using the standard phenol-chloroform method. Purified DNA (4 μl/100 μl) was used for qPCR amplification using the Eco Real-Time system (Illumina) and SYBR Premix Ex Taq II (Tli RNaseH Plus; TaKaRa) in a final reaction volume of 15 μL. Data were collected from three biological replicates; relative enrichment is expressed as percentage of input.

### Library construction, sequencing, and data analysis

For genomic resequencing of Kyoto, 1 μg genomic DNA was fragmented by shearing to an average fragment size of 330 bp using an Adaptive Focused Acoustics sonicator (Covaris, Woburn, MA, United States). After purification by a QIAquick PCR Purification Kit (Qiagen Valencia, CA, United States), a DNA library was constructed using a NEBNext DNA library prep master mix set for Illumina (New England Biolabs, Ipswich, MA, United States) and NEBNext Multiplex Oligos for Illumina (New England Biolabs). Briefly, the fragmented DNA was end-repaired, dA-tailed, and ligated with the NEBNext adapter. Size selection was conducted using AMPure XP magnetic beads (Beckman Coulter Brea, CA, United States) following the manufacturer’s instructions. The adapter-ligated DNA was amplified by eight cycles of high-fidelity PCR with an index primer. The PCR product was enriched using AMPure XP magnetic beads. Library quality and concentration were assessed using an Agilent Bioanalyzer 2100 (Agilent Technologies, Santa Clara, CA, United States). More precise concentrations of the libraries were determined by quantitative real-time PCR using a KAPA Library Quantification Kit (Kapa Biosystems, Wilmington, MA, United States). The paired-end library was sequenced by 200 cycles (2 × 100 bp) using the HiSeq 2500 (Illumina, San Diego, CA, United States). Raw reads were generated in FASTQ format using the conversion software bcl2fastq2 version 2.18 (Illumina).

For RNA sequencing, RNA was extracted from shoots samples using the TRI reagent (Sigma) and used to prepare libraries according to the protocol for the TruSeq RNA Sample Prep Kit v2 (Illumina). Libraries were subjected to paired-end sequencing for 75 cycles on a NextSeq500 (Illumina). After filtering the sequence reads with Trimmomatic version 0.39, they were aligned to the *Arabidopsis* public reference genome (TAIR10) using STAR version 2.7.0 with options –outFilterMultimapNmax 100 and –winAnchorMultimapNmax 100. For comparing transcript levels, reads aligned to genes were counted by featureCounts version 2.00 in the Subread package with default settings followed by normalization to counts per million (CPM). Differentially expressed genes and TEs were selected by adjusted *p*-values calculated using edgeR version 3.2.1 with default settings.

For bisulfite sequencing, an Illumina sequencing library (150-bp paired-end) was constructed using the post-bisulfite adapter tagging (PBAT) method (Miura et al., 2012) and sequenced at the OIST sequencing center. Reads were trimmed by Trimmomatic-0.33^1^ (Bolger et al., 2014) with the parameters: HEADCROP:10, SLIDINGWINDOW:4:20, and MINLEN:25. The remaining paired reads were mapped to the *Arabidopsis* Col-0 reference genome or the Kyoto genome retrieved from the 1001 Genomes Project (1001 Genomes Consortium, 2016) using Bismark (v0.12.1)^2^ (Krueger and Andrews, 2011) with the settings: –pbat -N 1 -X 500 –ambiguous -R 10 –un –score_min L,0,–0.6. Unmapped reads and single-end reads excluded from trimming were further mapped as single-end reads. Cytosine bases covering fewer than three reads were excluded; only uniquely mapped reads were used for further analysis. Methylcytosines were identified using a binominal test, with the bisulfite conversion rate estimated by mapping sequencing reads to the chloroplast genome.

Raw sequencing data for *cmt2* and *drm1/2* were downloaded from NCBI GEO (GSE39901). Adapter sequences and low-quality reads were trimmed using FASTP. Trimmed reads were aligned to the *Arabidopsis* TAIR10 genome with BSMAP 2.90 using the parameters: w100 -v2 -n1 -r1. Cytosine methylation was determined using BSMAP’s methratio.py script, which processes only unique reads and removes duplicates. Differentially methylated regions (DMRs) were detected using CGmapTools (v0.1.2). The threshold methylation difference for DMRs in each sequence was adjusted to 40, 20, and 10% for mCG, mCHG, and mCHH, respectively. DMRs were considered significant at *q* < 0.01. To identify DMRs contained within TEs, DMRs and TEs from TAIR10 were compared using BEDtools to identify overlaps.

### Data availability

Genome sequence data for Kyoto, RNA sequence analysis data, and whole-genome bisulfite sequence data were deposited in the DNA Data Bank of Japan (DDBJ) as DRA012845, DRA013053, and DRA012794, respectively.

## Results

### *ONSEN* is highly activated by heat stress in Kyoto

**FIGURE 1:**
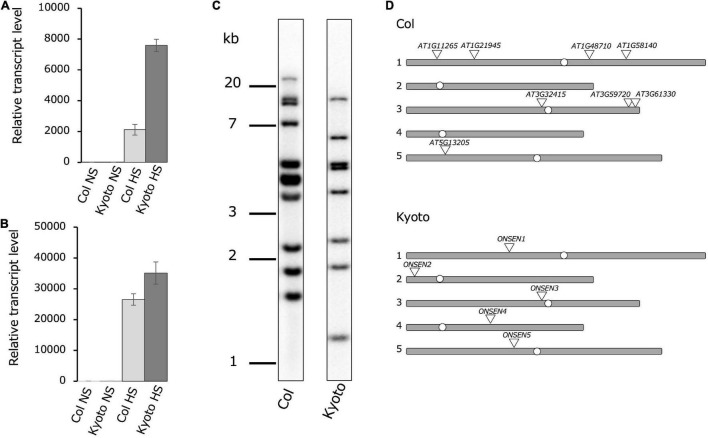
Comparison of *ONSEN* features in Col-0 and Kyoto. Real-time reverse transcription polymerase chain reaction showing relative *ONSEN* **(A)** and *HSFA2* **(B)** expression under heat stress (HS) and non-stress control (NS) conditions. All bars represent means ± SD from three biological replicates. Values shown are relative to those of Col-0 NS (set to 1). Relative *ONSEN* transcription levels were first normalized to 18S rRNA and then to Col-0. **(C)** Southern blot of *ONSEN* in Col-0 and Kyoto. **(D)** Chromosomal locations of *ONSEN* in subtypes based on sequencing of chromosomes 1–5.

To investigate differences in *ONSEN* activation among natural accessions of *Arabidopsis thaliana*, *ONSEN* transcription levels under heat stress were examined in 33 Japanese ecotypes (Supplementary Figure 1). The result showed that *ONSEN* was expressed at high levels in the ecotype Kyoto, approximately fourfold higher than that in the Col-0 ecotype (Figure 1A). Since previous studies have shown that HsfA2, a high-temperature stress-responsive transcription factor, regulates the transcriptional activation of *ONSEN* by high-temperature stress, the transcription levels of HsfA2 in Kyoto and Col-0 were measured; transcription was higher in the Kyoto subjected to heat stress than in Col-0 (Figure 1B). To determine *ONSEN* copy numbers, we compared the number of *ONSEN* bands obtained in Southern blotting of both ecotypes; eleven bands were detected in Col-0, while eight were detected in Kyoto (Figure 1C). The total number of bands detected by Southern blotting may reflect full-length *ONSEN* sequences and partial sequences.

To determine the genomic location of the full-length *ONSEN* copy, we used next-generation sequencing. There were five copies of the full-length *ONSEN* sequence in Kyoto, one copy each on chromosomes 1–5. Upon comparing these chromosomal positions in Col-0 with those in Kyoto, the copy on chromosome 3 near the centromere was conserved in the same position (Figure 1D). Since five copies of the full-length *ONSEN* sequence were detected in Kyoto and eight copies in Col-0, copy number alone does not explain increased *ONSEN* transcription in Kyoto.

### Heat stress causes genome-wide gene expression downregulation in Kyoto

**FIGURE 2:**
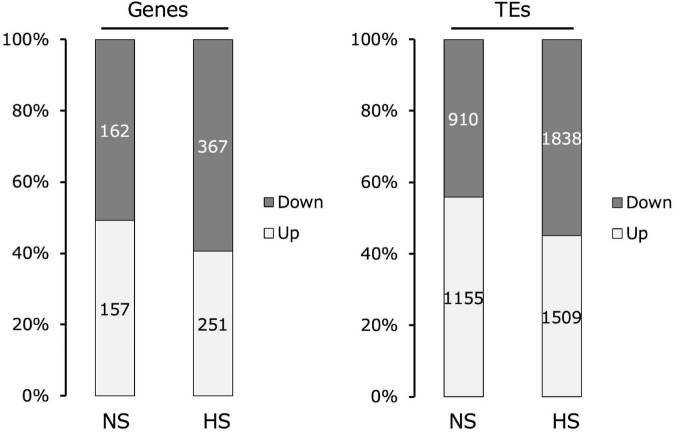
Comparison of changes in gene and transposable element (TE) expression in the transcriptomes of Kyoto compared with those of Col-0 seedlings. Transcripts with more than two-fold change are indicated.

Comparing the transcriptomes of Kyoto and Col-0 seedlings assembled using RNA-seq, Kyoto sequences with a more than two-fold change in transcript levels relative to Col-0 were classified into genes and TEs. The proportions of increased and decreased gene expression in Kyoto were roughly the same as in the non-stressed controls. Under high-temperature stress, the expression levels of nearly 60% of the genes decreased. In turn, the expression level of nearly 60% of the TEs increased under non-stress conditions, whereas only 45% of the TEs exhibited increased expression under high-temperature stress (Figure 2).

### DNA hypomethylation induces transcriptional derepression of *ONSEN* in Kyoto

**FIGURE 3:**
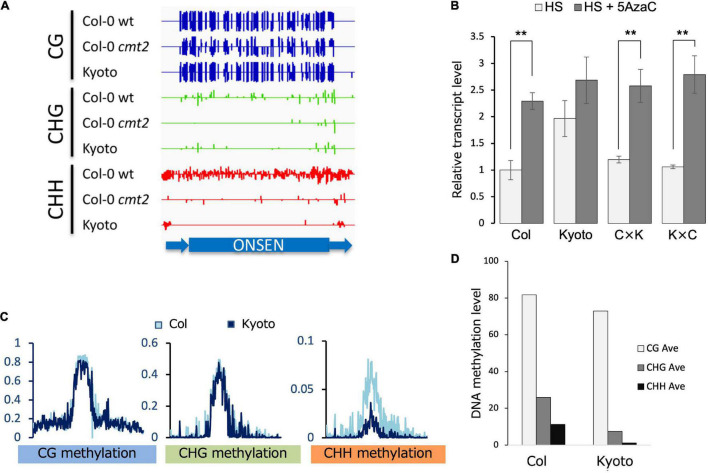
Transcription and methylation analyses. **(A)** DNA methylation in *ONSEN* sequences of Col-0, *cmt2*, and Kyoto. In the random mapping, the non-unique reads are mapped with reads evenly distributed among each copy, which is indicative of *ONSEN*’s overall methylation trend. **(B)** Transcript levels of *ONSEN* under heat stress (HS) and 5-aza-2’-deoxycytidine (5AzaC) treatment. Values represent means ± SD from three biological replicates. Asterisks mark statistically significant differences. **(C)** Genome-wide DNA methylation levels of transposable elements on NS. **(D)** Average *ONSEN* DNA methylation levels on NS.

Given that DNA methylation is a major factor in transposon repression, *ONSEN* methylation was analyzed. The methylation of CHG and CHH was lower in the *cmt2* mutant and Kyoto than in wild-type Col-0 in the absence of stress (Figure 3A). To determine the role of dominant or recessive factors in regulating *ONSEN* transcription in Kyoto, its transcription levels were analyzed in F1 hybrids of Kyoto and Col-0. The transcriptional level in the F1 hybrid was found to be similar to that of Col-0 (Figure 3B), suggesting that *ONSEN* expression in Kyoto is transcriptionally regulated in a recessive manner. To confirm that the upregulation in Kyoto was a consequence of reduced DNA methylation, the transcription levels of *ONSEN* in plants treated with the DNA methylation inhibitor 5AzaC were measured. *ONSEN* transcription in the F1 hybrid was increased by 5AzaC treatment (Figure 3B).

### Mutations in *CMT2* are responsible for the CHH hypomethylation in Kyoto

**FIGURE 4:**
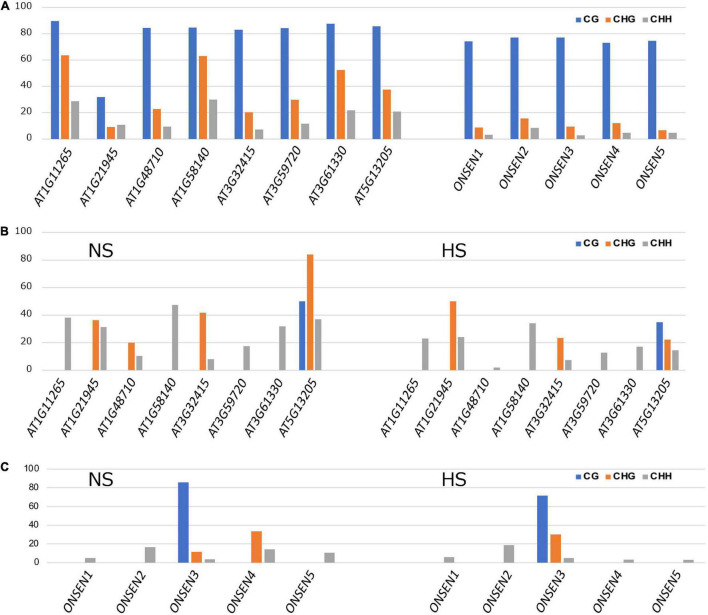
*ONSEN* methylation. **(A)** DNA methylation levels of *ONSEN* elements under non-stressed control conditions. **(B)** *ONSEN* LTR methylation levels in non-stress (NS) and heat-stressed (HS) Col-0. **(C)** *ONSEN* LTR methylation levels of individual in NS and HS Kyoto.

Comprehensive transposon DNA methylation analysis revealed that CHH methylation was reduced in Kyoto (Figure 3C). Also, the average CHH methylation level of *ONSEN* was lower in Kyoto than in Col-0 (Figure 3D). *ONSEN* CHH methylation levels varied between copies (Figure 4A). However, the levels were lower in Kyoto than in Col-0 for both NS and HS, whereas most of the methylation levels in *ONSEN* sequences after HS were reduced in Kyoto and Col-0 (Figures 4B,C).

**FIGURE 5:**
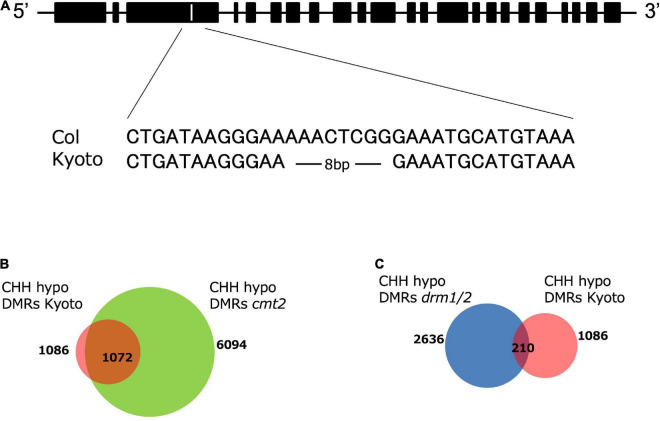
*CMT2* structure and DMR analysis. **(A)** Alignment of *CMT2* sequences from Col-0 and Kyoto. Kyoto has an 8-bp deletion. **(B)** Numbers of CHH hypo-DMRs in the *cmt2* mutant (green) and Kyoto (red). **(C)** Numbers of CHH hypo-DMRs in the *drm1/2* mutant (blue) and Kyoto (red).

To identify the genes responsible for high *ONSEN* expression in Kyoto, we crossed Kyoto with Col-0, obtained approximately 40 F2 lines expressing high and low *ONSEN* in heat-stressed F3, respectively, and extracted DNA from each group in batches. A library was constructed for each and sequenced. Analysis using Mitsucal (Suzuki et al., 2018), showed that SNPs were concentrated between the 6.6 to 10.7 Mb region of chromosome 4, containing the *CMT2* gene (Supplementary Figure 2). Since the loss of CMT2 caused a considerable decrease of CHH methylation across the genome (Zemach et al., 2013), we analyzed the Kyoto *CMT2* sequence and identified a deletion in the third exon (Figure 5A). DMR analysis showed that more than 98% of the hypo-CHH DMR regions of Kyoto and *cmt2* mutants overlapped (Figure 5B). To investigate the relationship between Kyoto and DRM1/2, which is another enzyme involved in CHH methylation in *A. Thaliana* (Du et al., 2012; Zemach et al., 2013), the overlap of the hypo-CHH DMR regions in Kyoto and *drm1/2* mutants was examined, revealing that only 19% of the regions overlapping (Figure 5C). These results suggest that the loss of CMT2 is likely involved in the high *ONSEN* expression in Kyoto.

### Repressive *ONSEN* histone modifications are decreased in Kyoto

**FIGURE 6:**
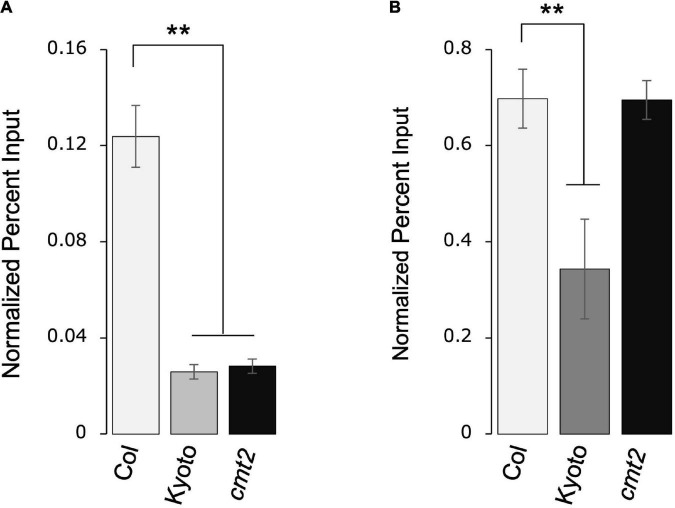
*ONSEN* histone modification. Accumulation of H3K9me2 **(A)** and H3K27me1 **(B)** in *ONSEN* under normal growth conditions. Data were quantified using chromatin immunoprecipitation-real-time polymerase chain reaction. Values represent means ± SD from three biological replicates. Asterisks mark significant differences from Col-0.

As histone modification regulates transposon activity, we measured the levels of repressive histone modifications H3K9me2 and H3K27me1 in Kyoto, Col-0 wild type, and Col-0 *cmt2*. Both H3K9me2 and H3K27me1 modifications were significantly decreased in Kyoto (Figures 6A,B). CMT2 is involved in the induction of H3K9me2; the decrease in H3K9me2 modification in the *cmt2* mutant was comparable to that in Kyoto, suggesting that the decrease in H3K9me2 in Kyoto is a consequence of the loss of CMT2. In contrast, the decrease in H3K27me1 occurred only in Kyoto; thus, a factor other than CMT2 is likely responsible for causing increased *ONSEN* expression in Kyoto.

### Kyoto centromere heterochromatin is dispersed by heat stress

**FIGURE 7:**
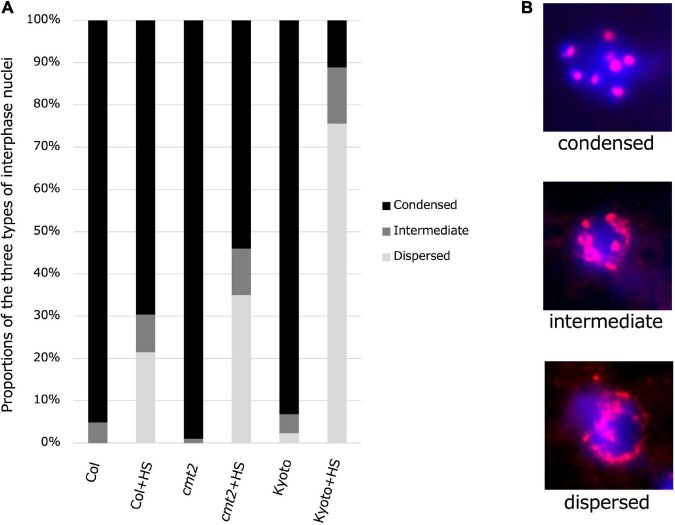
Condensatoin in interphase nuclei. **(A)** Proportions of three types of interphase nuclei observed in Col-0, *cmt2*, and Kyoto subjected to control or 24 h of high-temperature stress (HS24). **(B)** Representative images of types of interphase nuclei.

To evaluate the possibility that reduced methylation induces heat-induced relaxation of heterochromatin, cytological analysis of the interphase nuclei of Kyoto, Col-0 wild type, and Col-0 *cmt2* was performed, using hybridization with fluorescent probes for centromere repeat sequences (Figures 7A,B). The results showed that most interphase nuclei had condensed chromocenters in the untreated state. However, after high-temperature treatment, the percentage of cells with diffused chromocenters was increased in Kyoto than in Col-0 wild type (Figure 7A). Interestingly, chromocenter diffusion after high-temperature treatment in Kyoto was more significant than in the Col-0 *cmt2* (Figure 7A).

## Discussion

Although TEs are present in most organisms, the diversity of their modes of activation remains unknown. Here, we investigated the diversity of TE transcriptional activity induced by high temperatures in *A. thaliana*, finding that *ONSEN* transcription was significantly upregulated in the Japanese ecotype Kyoto. Multiple *Arabidopsis* ecotypes exist worldwide, with *ONSEN* copy number correlated with the annual habitat temperature range (Quadrana et al., 2016), indicating that *ONSEN* provides a compelling case for a causal relationship between climate and TE mobilization. As its name suggests, the Kyoto ecotype is present near Kyoto. Thus, it is of interest to determine if a correlation between geographical factors in Japan and *ONSEN* transcription levels exists. However, increased *ONSEN* transcription was not observed in other Japanese ecotypes, and no clear explanation exists for the increase in *ONSEN* transcription levels only in Kyoto. When considering the transcription of TEs in the host genome, the total transcription level is likely affected by the transposon copy number. A comparison of *ONSEN* copy number among ecotypes showed that none had a markedly high copy number, with a range of one to tens of copies. In addition, when the copy number of a transposon reaches a certain threshold, the host induces epigenetic regulation to repress transcription. In fact, the high *ONSEN* transcription found in this study is not due to copy number differences, but rather the release of transcriptional silencing *via* CHH methylation. Our data suggest that increased *ONSEN* transcription in Kyoto is likely caused by a loss-of-function mutation in the gene encoding the plant-specific methyltransferase CMT2.

Previous reports had shown that CHH methylation in *ONSEN* is mediated primarily by CMT2. The *cmt2* mutant showed reduced CHH methylation, but not *drm1 drm2* mutants (Nozawa et al., 2021). Similarly, a dramatic decrease in CHH methylation at *ONSEN* in *cmt2 cmt3*, but not in the *drm1 drm2 cmt3* triple mutant, further confirmed that CMT2 catalyzes CHH methylation at *ONSEN* (Nozawa et al., 2021). Recent studies have also shown that DNA methylation is required for histone H1-mediated suppression of *ONSEN* in the heat stress response; DNA methyltransferase inhibitors have been shown to suppress the increased expression of *ONSEN* in heat-treated *h1* mutants. In addition, the expression of *ONSEN* was found to be incleased in heat-treated *cmt2* mutants (Liu et al., 2021).

The natural *CMT2* mutant allele detected in Kyoto was different from the one with the premature stop codon in the first exon (*CMT2*_*STOP*_) reported by Shen et al. (2014). They found that accessions with the *CMT2*_*STOP*_ allele had a small (1%) average reduction in CHH methylation across the TE-body compared to those of wild-type CMT2 (*CMT2*_*WT*_). A more detailed analysis revealed that the difference was mainly due to two of the 16 *CMT2*_*stop*_ accessions, Kz-9 and Neo-6, showing a TE-body CHH methylation pattern similar to the *cmt2* knockout. The methylation patterns were also shown to be more heterogeneous between natural accessions than Col-0 for both the *CMT2*_*STOP*_ and *CMT2*_*WT*_ alleles. Furthermore, the contribution of the CMT2-independent CHH methylation varied among natural accessions. Although it is unclear why not all *CMT2*_*STOP*_ accessions behave like null alleles, the variation in CHH methylation among natural accessions suggests that CMT2-independent pathways, such as the RdDM pathway, may compensate for the lack of CMT2 at these loci by segregating polymorphisms. Alternatively, *CMT2*_*STOP*_ alleles may not be nulls due to read-through of stop codons. Similarly, Sasaki et al. (2019) also identified a natural mutant allele of *CMT2*, but none of these seem to be present in the Kyoto. Indeed, the *cmt2b’* allele (Sasaki et al., 2019), had a lesser effect on CHH methylation than the Kyoto, which was nearly identical to the expression of the experimental *cmt2* knockout allele. These data suggest that the spontaneous *cmt2* mutation in Kyoto may be a novel allele and may represent a significant recurrence of *cmt2* mutations in nature.

It has been shown that *cmt2* mutants are more heat tolerant (Shen et al., 2014), suggesting that genetic regulation of epigenetic modifications through differential allelic plasticity to temperature stress may be a mechanism underlying natural adaptation to variable temperatures. Since the mutation was also found in the *cmt2* allele of Kyoto, stress tolerance at high temperatures was investigated; however, no particular stress tolerance was observed in comparison with the other ecotypes (Supplementary Figure 3). Therefore, the reduction in CHH methylation present in the Kyoto may not have been sufficient to confer stress tolerance. It is possible that stress responses *via* changes in other epigenetic modifications within the Kyoto ecotype are responsible for differences from other ecotypes and prevented Kyoto from exhibiting stress tolerance. Moreover, the level of H3K27me1 modification in Kyoto was found to be considerably lower than that in the *cmt2* mutant of Col-0. Thus, another possibility is the presence of factors in Kyoto that have a counteracting effect on high-temperature stress tolerance, as shown for other *cmt2* mutants. To confirm this hypothesis, the responsible factors must be mapped and identified.

The level of H3K9me2, which forms a feedback loop with CHH methylation, was reduced in Kyoto to the same extent as it was in the *cmt2* mutant. This is consistent with the findings of reduced CHH methylation in Kyoto. Interestingly, while the level of H3K27me1 was also reduced in Kyoto, the elements responsible are currently unknown; however, it is highly likely that there is diverse epigenetic regulation of the ecotype. Moreover, although the Kyoto was not heat-tolerant, interphase heterochromatin contained a higher number of cells that were more likely to diffuse than those in Col-0 *cmt2* and Col-0 wild type. The differences in the degree of chromocenter condensation in interphase nuclei may reflect differences in the level of H3K27me1 modification. Although the associated biological significance requires further investigation, there were no remarkable phenotypic changes throughout the plant after heat stress. Thus, the relevance of this epigenetic polymorphism to responses to other environmental stresses is of great interest.

In this study, we examined ecotype-dependent transcriptional regulation using transposons that are activated under heat stress; however, in the future, it will be necessary to investigate the diversity of transcriptional regulation under additional stresses. CHH methylation is mainly localized to TE sequences. Therefore, reducing CHH methylation by a loss-of-function CMT2 would significantly impact the TE sequence, affecting the transcription of transposons that are activated by stress, such as *ONSEN*. In addition, gene expression in the vicinity of transposons would also be affected by the hypomethylation caused by deficiencies in CMT2. The fixation of polymorphisms in epigenetic regulators within natural populations, such as Kyoto, may adapt host organisms to adjust to different growing environments.

One limitation of the current study is that *Arabidopsis* ecotypes likely are adapted to their environments, thus, ecotypes collected relatively recently may have only existed for a short time and may not, therefore, directly reflect the growing environment of the site where they were collected. Therefore, it is necessary to make multifaceted comparisons based on genomic structural differences, conservation of transposon sequences, and similarities in chromosomal positions and copy number. Furthermore, it should be noted that the ecotypes from regions adjacent to Kyoto, Japan were not always observed in the same way, so the names of *Arabidopsis* ecotypes do not always reflect their local environments.

## Data Availability Statement

The datasets presented in this study can be found in online repositories. The names of the repository/repositories and accession number(s) can be found below: The genome sequence data for Kyoto, RNA sequence analysis data, and whole-genome bisulfite sequence data were deposited in the DNA Data Bank of Japan (DDBJ) as DRA012845, DRA013053, and DRA012794, respectively.

## Author Contributions

KN and SM coordinated this work. HS performed the bisulfite-sequencing analysis. YI performed the ChIP analysis. TS, HT, and KT performed the genome-wide analysis using next-generation sequencing. NO performed the high-temperature stress tolerance experiments. KN and XN performed the cytological experiments. KN, AK, and HI wrote the manuscript. All authors have read and agreed to the submission of the manuscript.

## Conflict of Interest

The authors declare that the research was conducted in the absence of any commercial or financial relationships that could be construed as a potential conflict of interest.

## Publisher’s Note

All claims expressed in this article are solely those of the authors and do not necessarily represent those of their affiliated organizations, or those of the publisher, the editors and the reviewers. Any product that may be evaluated in this article, or claim that may be made by its manufacturer, is not guaranteed or endorsed by the publisher.

## Footnotes

1
http://www.usadellab.org/cms/?page=trimmomatic

2
https://www.bioinformatics.babraham.ac.uk/projects/bismark/
