# Supplementary material for: Epigenetic regulation of ecotype-specific expression of the heat-activated transposon ONSEN
Source: Front Plant Sci. 2022 Jul 18;13:899105. doi: 10.3389/fpls.2022.899105 (PMC9340270; doi:10.3389/fpls.2022.899105)
Supplement: Supplementary file 1 [file Table_1.pdf]

Sup. Table 1 Primer list

Name	Sequence	Additional Information
ONSEN-F	TAATGTTCCCTTCCAAGTCCC	Southern probe
ONSEN-R	GCTTGTAATGACCCAAGAAGT	
ATH180F	GATCAAGTCATATTCGACTC	FISH probe
ATH180R	GTTGTCATGTGTATGATTGA	
COPIA78-4129F_RT	CCACAAGAGGAACCAACGAA	RT-qPCR, ChIP-qPCR
COPIA78-4300R_RT	TTCGATCATGGAAGACCGG	
18Sr-FW	CGTCCCTGCCCTTTGTACAC	RT-qPCR
18Sr-RV	CGAACACTTCACCGGATCATT	
TSP1-F	GAACTCATGGATACCCTAAAATAC	
TSP1-R	CTCTACCCTTTGCACTCATGAATC	
MULE qRT-PCR Fw	GGCACTTCAATTGTGCTTTTTCCT	
MULE qRT-PCR Rv	GATACTTGTTGACAAGTGTTTAGCAAGCC	
HsfA2_qPCR_F	TGGGATTCTCATAAGTTCTCAACA	
HsfA2_qPCR_R	TGGATCAATCTTTCTGAATCCAT	
MULE pri3F	TCAGGCACTTCAATTGTGCT	ChIP-qPCR
MULE pri3R	GACAAGTGTTTAGCAAGCCAAC	
ACTIN2 pri3F	GGCTTAAAAAGCTGGGGTTT	
ACTIN2 pri3R	TTGTCACACACAAGTGCATCA	
kyoto_cmt2_F	GTAGGGACAGAACGTTAGGA	Genotyping PCR
kyoto_cmt2_R	TACATGCATTTCCCGAGTT	
Kyoto_CMT2_R	CTACGTTTGTTCTGCTCTTG	
