# Supplementary material for: Epigenetic regulation of ecotype-specific expression of the heat-activated transposon ONSEN
Source: Front Plant Sci. 2022 Jul 18;13:899105. doi: 10.3389/fpls.2022.899105 (PMC9340270; doi:10.3389/fpls.2022.899105)
Supplement: Supplementary file 2 [file Presentation_1.pdf]

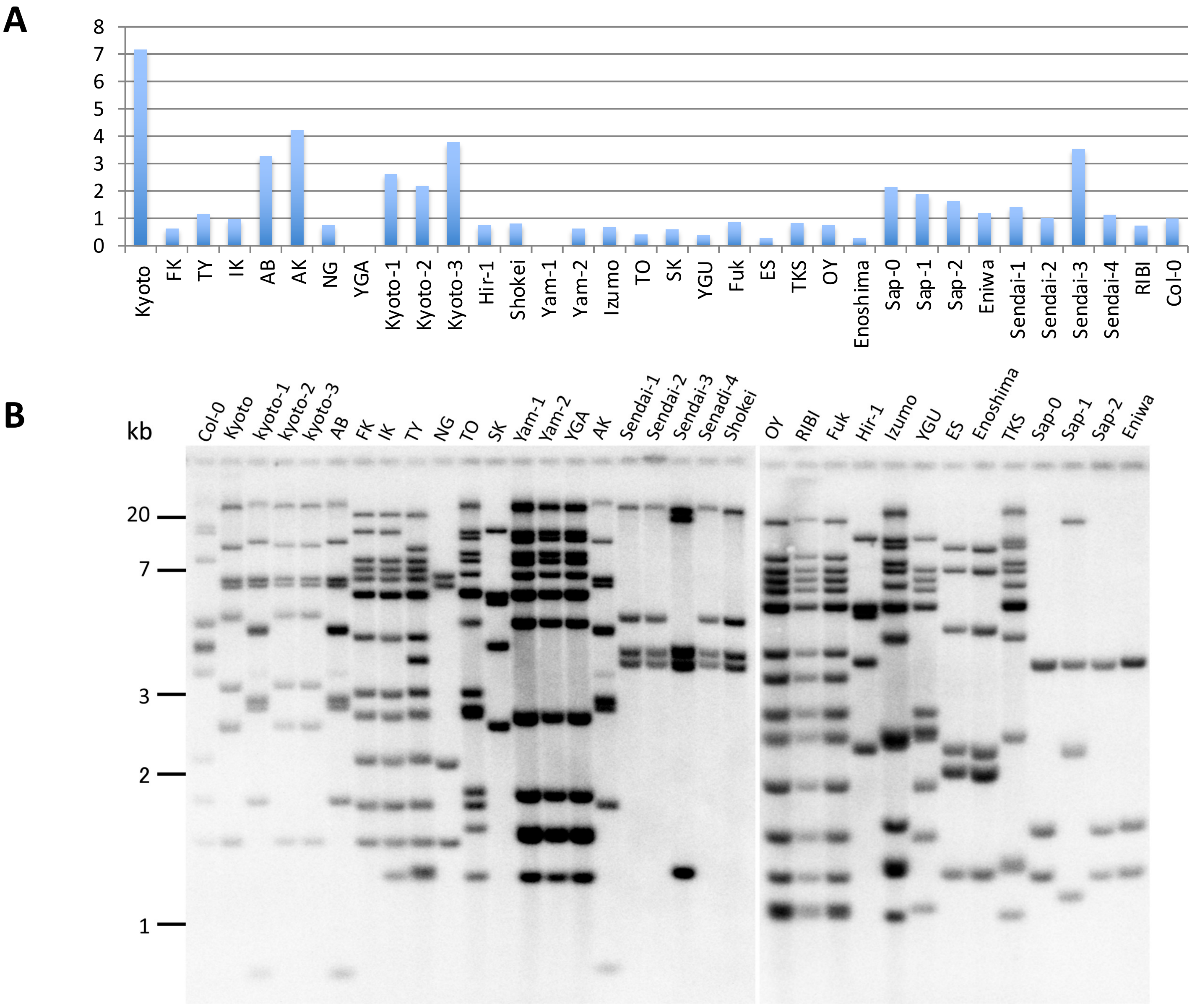

Sup. Fig.1 Level of *ONSEN* expression and number of copies in Japanese ecotypes **(A)** Transcription level of *ONSEN* in Japanese ecotypes of *Arabidopsis thaliana*. **(B)** Southern blotting of *ONSEN* in the Japanese ecotypes.

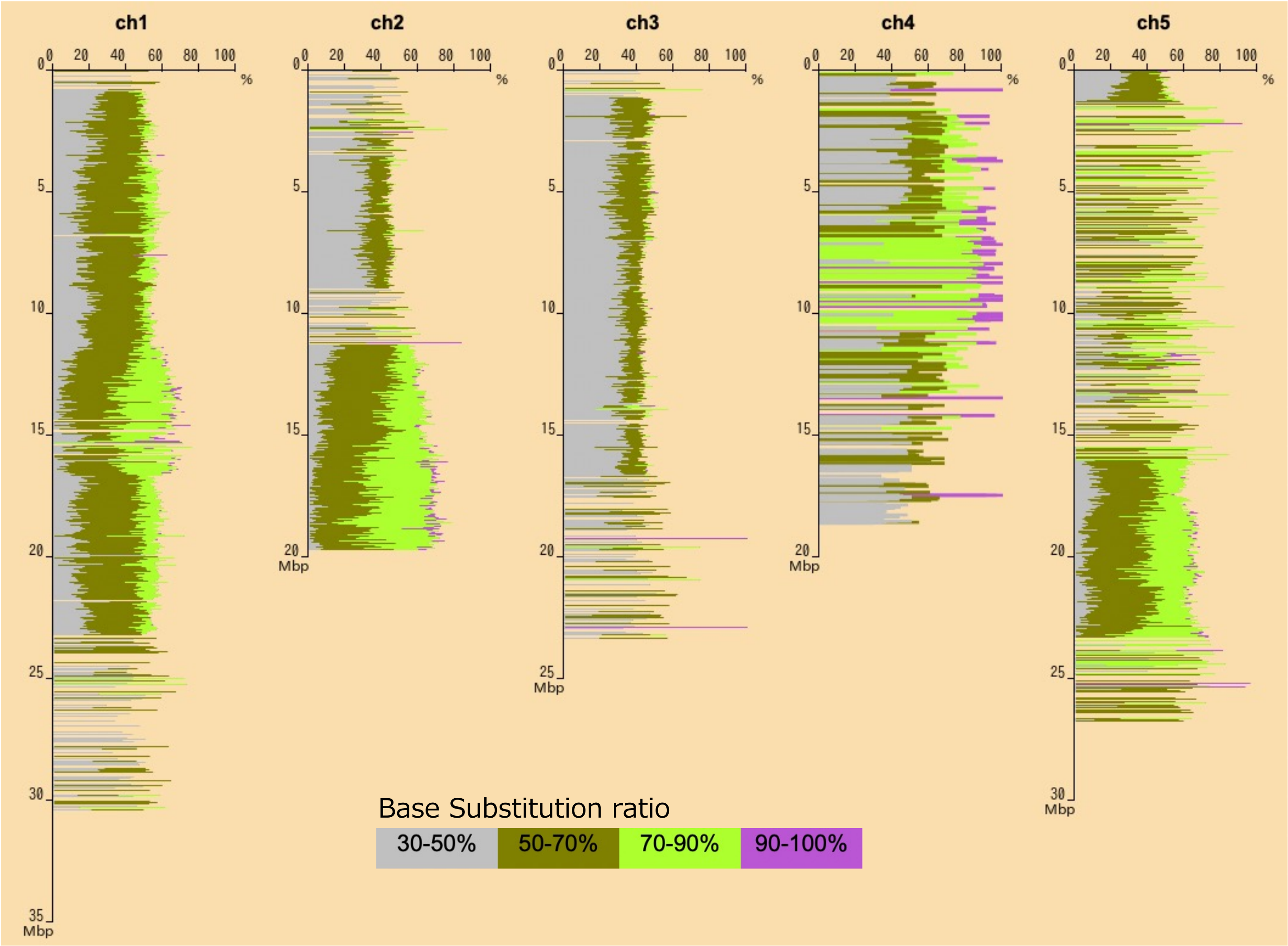

Sup. Fig.2 Chromosome mapping displaying nucleotide substitution rate using Mistucal.

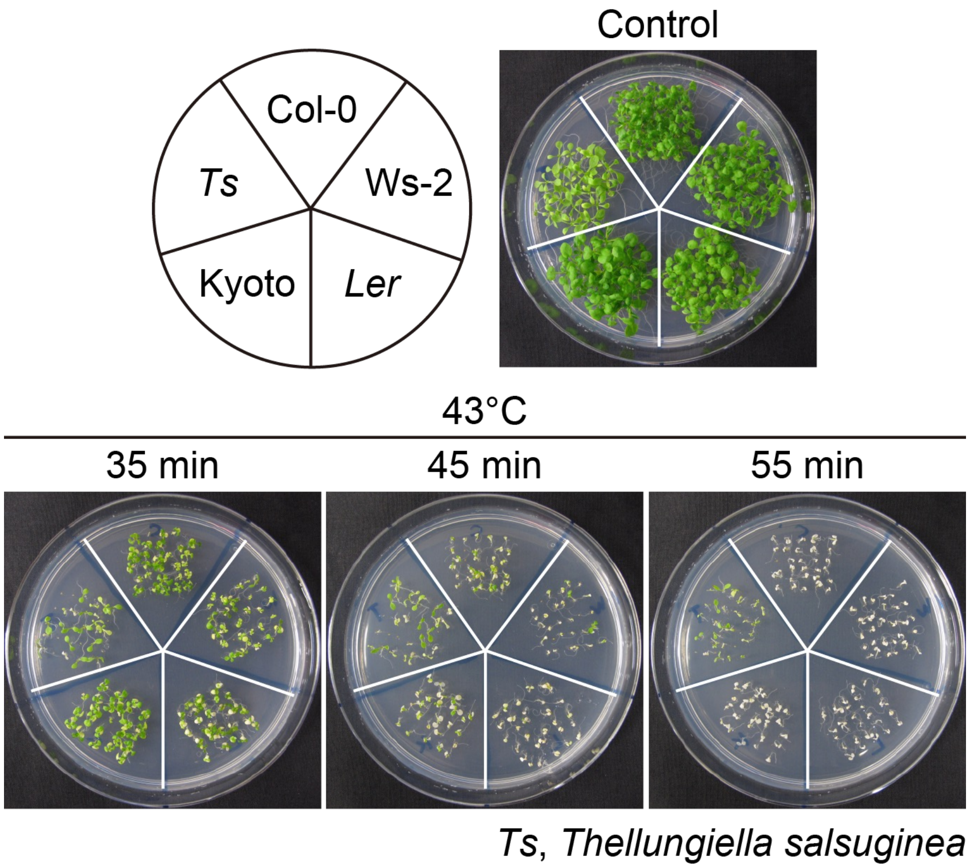

Sup. Fig.3 High temperature tolerance test. *Ts* was used as a positive control for high temperature tolerant plants.
